# Does radiographic imaging for spinal pain in patients receiving chiropractic care change patient outcomes? A pilot randomised controlled trial

**DOI:** 10.1186/s12998-026-00662-y

**Published:** 2026-07-02

**Authors:** Hazel J. Jenkins, Mark Hancock, Aron Downie, Anika Young, Craig Moore, Stephney Whillier, Simon D. French

**Affiliations:** 1 Department of Chiropractic, Macquarie University, Sydney, Australia; 2 Macquarie University Spinal Pain Research Centre, Macquarie University, Sydney, Australia; 3 School of Health Sciences and Nursing, Macquarie University, Sydney, Australia

**Keywords:** Spinal pain, Radiographic imaging, Pilot randomised controlled trial, Chiropractic, Spinal manipulative therapy

## Abstract

**Background:**

Imaging does not improve the management of non-specific spinal pain in randomised trials (RCTs) conducted in medical care settings. No RCTs have been performed to assess the benefit of imaging in chiropractic clinical settings, where it is postulated imaging may identify contraindications to, or inform application of, spinal manipulation. We aimed to assess the feasibility of performing a large-scale RCT to test whether obtaining spinal radiographs for patients receiving chiropractic care for spinal pain changes patient outcomes.

**Methods:**

Chiropractor and patient recruitment occurred from February 2021 to June 2022. Chiropractors from Sydney, Australia screened consecutive patients for inclusion in the pilot RCT. The key inclusion criteria were being an adult with a new episode of spinal pain and uncertain need for radiographs (i.e., no definitive indications or contraindications for imaging). Participants were randomised to receive spinal radiographs, or not. Clinical outcome data were collected at baseline, 2-, 6-, and 12-weeks. Chiropractors participated in semi-structured interviews regarding their experience in the trial. Feasibility outcomes were reported descriptively, including: proportion of chiropractors and patients agreeing to be involved, patient cross-over, loss to follow-up, and acceptability.

**Results:**

Eleven chiropractors screened 135 patients (mean 12, SD 17.0, range 0–56), with 33/135 (24%; 95%CI 18–32) meeting inclusion, and 20/33 (61%; 95%CI 44–75) participating in the trial (10 randomised to each group). Participating patients tended to be young and have low spine-related disability. 30% of those randomised to radiography did not receive imaging (95%CI 11–60; 3/10). Conversely, 20% (95%CI 6–51; 2/10) of those from the no radiography group received imaging. Follow-up data were collected from 90% of patients (95%CI 70–97) at 2-weeks and 75% (95%CI 53–89) at 12-weeks. Complete follow-up data for patients was provided by all chiropractors at 2- and 6-weeks and 16/20 (80%; 95%CI 58–92) at 12-weeks. Patient reported outcomes improved in both groups, with infrequent and mild adverse events. Chiropractors thought the research question important but highlighted key feasibility issues impacting patient recruitment.

**Conclusion:**

Progression criteria to conduct a large-scale RCT were largely met; however, suitable strategies would be required to ensure feasibility of patient recruitment and reduce cross-over between groups.

**Supplementary Information:**

The online version contains supplementary material available at https://doi.org/10.1186/s12998-026-00662-y.

## Background

There is a long history in the chiropractic profession of the use of spinal radiographic imaging and spinal manipulative therapy. From as early as 1910, chiropractors have reported using spinal radiographs as a diagnostic tool, and a number of chiropractic technique protocols were developed around radiographic analysis [1]. Historically, radiographic imaging was primarily used by chiropractors to visualise and assess spinal alignment, and thus direct the application of spinal manipulative therapy [2]. Although spinal manipulative therapy remains a common technique performed by chiropractors [3, 4], evidence for the effectiveness of imaging to guide spinal manipulative technique application is limited [5, 6].

In recent years, guidelines for imaging use advise against routine spinal imaging. Instead imaging is recommended when there is suspicion of serious pathology (e.g. cancer, fracture, or infection) and a change in management may be needed (e.g., referral for medical care) [5, 7]. When and how imaging should be used within chiropractic practice remains controversial, with academic debate through journal papers [5, 8, 9] and panel discussions at professional conferences [10]. As a result, chiropractors report a wide variance in both spinal radiography utilisation rates (8% to 84%) [5] and reasons for imaging referral (e.g., assessment of spinal alignment, screening for contraindications for spinal manipulation) [11–13].

The inappropriate use of spinal radiography is associated with potential risks to patients. These include radiation exposure, false reassurance where radiography lacks sufficient sensitivity to detect clinically important pathology (e.g., early cancer, infection, or osteoporosis [14, 15]), overdiagnosis of incidental findings that may lead to patient anxiety or further unnecessary treatments or investigations, and inefficient use of healthcare resources [5, 16–18]. In contrast, the clinical benefits of imaging use, when serious pathology is not suspected, remain uncertain [5, 6, 19]. In general medical practice, two randomised controlled trials (RCTs) have demonstrated that the use of routine radiographic imaging does not improve clinical outcomes for people with non-specific low back pain [20, 21]. A matched cohort study performed in patients presenting for chiropractic care for low back pain similarly found no difference in clinical outcomes (pain, disability) or satisfaction with care when participants received or did not receive radiographic imaging [22]. However, no RCTs have specifically assessed the effect of obtaining radiographic imaging on patient outcomes or adverse events within a chiropractic setting, where patients also receive spinal manipulative therapy [6]. In addition, the effect of imaging use to reduce adverse events associated with treatment has not been assessed in either general medical or chiropractic clinical settings. Concerns from chiropractors that they may miss important contraindications for spinal manipulation or perform sub-optimal spinal manipulation, thus resulting in poorer patient outcomes or more adverse events [23], cannot be addressed without this research.

Performing a RCT to assess the effect of radiographic imaging use on patient outcomes has several associated challenges. Chiropractors need to be able to identify situations of clinical equipoise related to the decision to refer for not refer for spinal radiographs. In other words, identifying situations where the chiropractor does not know whether referring for radiographs or not will provide better treatment and are therefore willing to randomise patients to receive, or not receive, spinal radiographs. Chiropractors who feel strongly about the use of radiographic imaging (either for or against) may not be willing to participate. Patients may also have strong beliefs regarding the need for imaging to obtain optimal management [24, 25] and may not be willing to be randomised. These challenges may significantly impact the success of a large scale, resource-intensive RCT. Hence, the feasibility of conducting this research needs to be assessed.

The primary aim of this pilot study was to assess the feasibility of performing a large-scale RCT. A fully powered trial will test whether radiographic imaging for spinal pain, for patients receiving spinal manipulative therapy, effects patient outcomes, including the frequency or degree of adverse events. The aims of this pilot study are to assess: (1) chiropractor and patient recruitment; (2) compliance with the randomisation schedule; (3) compliance with study processes and acceptability of the trial; and (4) preliminary findings for patient outcomes that may affect trial feasibility (health outcomes, adverse events, satisfaction with care).

## Methods

### Study design

A pilot RCT was performed to assess the feasibility of performing a large-scale trial to assess the effectiveness of radiographic imaging to change health outcomes or adverse events in people receiving chiropractic care (including spinal manipulative therapy) for spinal pain. Ethics approval was provided by Macquarie University Human Research Ethics Committee, reference number: 52020912721836. The trial was prospectively registered through the Australia and New Zealand Clinical Trials Register (ANZCTR), Trial ID: ACTRN12621000087853 (date registered 1st February, 2021). The trial was reported in accordance with the CONSORT 2010 extension to randomised pilot and feasibility trials [26] (checklist available in Supplementary file 1).

### Chiropractor recruitment

Chiropractors working within Sydney, Australia (or immediate surrounds) were recruited through professional networks and recommendations from participating chiropractors. Recruitment commenced on 15 February, 2021. To participate, chiropractors had to self-report regularly treating people with spinal pain, commonly using spinal manipulative therapy, and referring between 20 and 80% of new patients for radiographic imaging. Responses were based on self-reflection, not clinical audit, and chiropractors were asked to reflect on what they considered their typical practice. Chiropractors typically referring less than 20% or more than 80% of new patients for radiography would be less likely to have clinical equipoise related to the decision to refer for imaging, as required for patient randomisation (i.e., need to be willing for the patient to be randomised to receive or not receive radiography). A member of the research team met with any interested chiropractors to check inclusion criteria, explain the study, and obtain consent to participate. Chiropractors were informed that participation would include patient recruitment, completion of baseline and follow-up surveys for each patient, and participation in a semi-structured interview. Participating chiropractors received AUD$50 reimbursement per recruited patient to compensate them for their time.

### Patient recruitment

Consecutive patients with a new episode of spinal pain were informed of the trial by chiropractors at the end of the new patient assessment. To be included, patients needed to be presenting with a new episode of spinal pain, defined as no prior episode of similar pain (with greater than two out of ten on a numerical pain score) in the last month, and be able to read and understand English. In addition, the chiropractor needed to be intending to use spinal manipulative therapy as part of patient management and have clinical equipoise in the decision to refer the patient for radiographic imaging (i.e., no absolute indications [e.g., red flags for suspicion of underlying pathology] or contraindications for radiography [e.g., pregnancy] and clinical uncertainty as to whether radiography would result in better outcomes). Chiropractors were asked to use evidence-based guidelines to determine whether radiography was indicated or not, as summarised in a review of radiography use for chiropractors [5]. Patients were excluded if they were younger than 18 years of age, pregnant, had already received recent spinal imaging, or the chiropractor suspected serious underlying pathology that would require further investigation or referral (e.g., cancer, infection, fracture, cauda equina syndrome, myelopathy, progressive neurological symptoms, chest or abdominal pathology). Patients meeting inclusion criteria and interested in participating in the trial were contacted by the research team to provide more information about the trial, obtain consent, and perform randomisation.

To limit the trial processes causing delay in obtaining imaging, all patients who expressed interest in participating in the trial (prior to providing consent and being randomised) were provided with a radiographic imaging referral for the spinal region/s of relevance to their presentation (as deemed appropriate by the treating chiropractor). The referral forms were provided in a sealed envelope, and the patient was asked to only open and use the referral form if they were randomised to the intervention group. In this way the patient could obtain imaging as soon as randomisation was performed, without the need for another visit to the chiropractor to obtain the referral form.

### Randomisation

Randomisation of participating patients was performed to allocate them to either receive radiographic imaging (intervention group), or not (control group). Computer generated random number lists were created and concealed allocation was performed through REDCap [27, 28] after the patient consented to participate. The generation of the random number lists was performed centrally by a researcher not involved in participant recruitment. Blinding of the patient and chiropractor was not possible; however, allocation was only revealed after baseline questionnaires were completed by both the patient and chiropractor.

### Intervention

Patients allocated to the intervention group were asked to use the spinal radiographic imaging referral they had been provided with as soon as possible. All imaging referrals were made under the Australian Medicare system, and patients did not have any out-of-pocket expenses. Patients allocated to the intervention group who did not obtain imaging were recorded as a cross-over between groups. Any further treatment provided, including the application of spinal manipulative therapy, was determined between the chiropractor and patient and was not dependent on whether the imaging was performed.

### Control

Patients allocated to the control group were asked to not use the radiographic imaging referral that they had been provided with. Any further treatment, including the application of spinal manipulative therapy, was provided as determined between the chiropractor and patient. If it was subsequently determined that radiographic imaging was required, a new referral would be provided by the chiropractor. Use of a subsequent imaging referral was recorded as cross-over between groups.

### Study processes

After consenting to participate, chiropractors completed an initial online questionnaire and attended a 30-minute training session outlining the study procedures. The questionnaire included demographic questions: age, sex, years in practice, place of education; and self-recall questions regarding radiographic imaging use: frequency of referral, location of referral (in-house/radiology centre), common reasons for imaging referral, and beliefs about imaging use (i.e., beliefs about the importance of imaging in the management of spinal pain). Clinician training included the processes to screen patients for inclusion in the trial, how to identify clinical equipoise in the need for spinal radiographs, and scripts to help explain the trial to the patient (including the identification of clinical equipoise) and ask for consent to be contacted by the research team.

Chiropractors were asked to assess consecutive new patients for inclusion during new patient consults and complete an online checklist for each patient regarding whether the patient met inclusion criteria, was invited to receiving further study information, and consented to be contacted by the researchers. The reasons for not screening a patient or inviting them to participate were also recorded. Trial recruitment was interrupted due to the COVID-19 pandemic. Chiropractors were asked to assess patients for recruitment to the study through two study periods from March to July, 2021 and March to June, 2022. A second training session to remind chiropractors of the study procedures was performed prior to the start of the second study period. At the end of the second study period all chiropractors were asked to attend a face-to-face or online semi-structured interview with one of the research team regarding their experiences in the study.

Participating patients completed an online baseline questionnaire (prior to receiving randomisation allocation) and follow-up questionnaires at 2-weeks, 6-weeks, and 12-weeks post-randomisation. The baseline questionnaire collected demographic and clinical information including age, sex, educational background, occupation; questions about their pain presentation: numerical pain score, disability questionnaire (Roland Morris/Neck disability index), previous pain history, history of imaging, history of previous treatment; and questions about imaging beliefs and expectations. The follow-up questionnaires collected clinical information including numerical pain score, disability (Roland Morris/Neck disability index), patient satisfaction, experience of adverse events (defined as any new unwelcome symptoms or an increase in presenting symptoms [29]), care-seeking, and whether radiographic imaging was obtained.

For each participating patient the chiropractor was asked to complete online questionnaires about the participant at baseline (pre-randomisation) and at 2-weeks, 6-weeks, and 12-weeks post-randomisation. The baseline questionnaire collected clinical information about the participant including the presence of red flags, differential diagnosis, and proposed treatment modalities. The follow-up questionnaires collected information on adverse events, treatment modalities applied (spinal region and type), and whether a subsequent radiographic imaging referral had been provided.

### Data collection

All quantitative data were collected and managed using REDCap electronic data capture tools hosted at Macquarie University [27, 28].

#### Chiropractor and patient recruitment (aim 1)

To assess the recruitment of chiropractors we recorded the number of chiropractors who: were approached; were screened; met inclusion; and consented to participate. Reasons for non-participation were collected as free-text responses. To assess the characteristics of recruited chiropractors we collected demographic data and information on their use of radiographic imaging.

To assess the recruitment of patients we recorded the number of patients: screened; meeting inclusion criteria; invited to participate in the study; and who consented to participate. Reasons for non-participation were collected as free-text responses. To assess the characteristics of recruited patients we collected demographic and clinical data and information on their beliefs about the use of radiographic imaging.

#### Compliance with the randomisation schedule (aim 2)

We recorded the number of patients randomised to each group and whether the patient obtained radiographic imaging during the study (yes/no). If the patient obtained or didn’t obtain imaging out of alignment with the randomisation schedule (cross-over) we recorded the reason as a free-text response.

#### Compliance with study processes and acceptability of the trial (aim 3)

We collected data on the number of chiropractors who consented to participate in the study who subsequently: attended the training session; screened patients for inclusion; and recruited patients to the study. Completion rates of baseline and follow-up questionnaires by both patients and chiropractors were also collected.

Barriers and enablers to complying with the study processes and the acceptability of randomising patients to receive or not receive x-rays were assessed in the post-study interview. The interview included open-ended questions on the chiropractors’ experience in the study, their ability to comply with the study processes, and any unanticipated advantages or disadvantages of being involved in the study. The interview guide is available in Supplementary file 1. Interviews were performed online using Microsoft Teams and were recorded and transcribed by one of the research team (AY).

#### Preliminary findings (aim 4)

At each follow-up time-point we collected data from the chiropractors and patients on adverse events, pain intensity, disability, and satisfaction with care.

### Sample size

We aimed to recruit 10 chiropractors and 50 patient participants (25 per group). Pilot studies are designed to identify problems that would need to be resolved before, or that might preclude, a full-scale trial. A sample size of 50 patients provides 95% confidence for detecting problems occurring in at least 6% of the population [30]. In the event that this target was not met, a minimum of 20 participants (10 per group) would provide 95% confidence for detecting problems occurring in at least 15% of the population [30].

### Data analysis

#### Chiropractor and patient recruitment (aim 1)

The proportions of chiropractors and patients meeting each stage of the recruitment process were calculated as percentages with 95% confidence intervals (95%CI). Counts of people not meeting individual inclusion criteria were calculated and content analysis was used to describe the reasons for not wanting to participate in the study. Demographic characteristics, clinical characteristics, imaging beliefs, and imaging use were described to assess the characteristics of recruited chiropractors and patients. Baseline characteristics of chiropractors were stratified by high, low, or no recruitment, with high recruitment defined as recruiting four or more patients into the study.

#### Compliance with the randomisation schedule (aim 2)

The proportion of cross-over between intervention and control groups was calculated with 95%CI and reasons for cross-over were described.

#### Compliance with study processes and acceptability of the trial (aim 3)

The proportions of chiropractors and patients completing each stage of the study processes, including completion of questionnaires, were calculated as percentages with 95%CI. Qualitative interview data were coded in NVivo 12 plus by one of the researchers (AY). Data were analysed using framework thematic analysis [31] to describe barriers and enablers to complying with the study processes. The initial thematic framework was determined by consensus between two of the researchers (HJ and AY), with final analysis discussed and agreed upon by the research team.

Acceptability was assessed through qualitative interview data analysed using framework thematic analysis [31] to describe chiropractors’ opinions on the process of randomising patients to receive x-rays. The initial thematic framework was determined by consensus between two of the researchers (HJ and AY), with final analysis discussed and agreed upon by the research team.

#### Preliminary findings (aim 4)

Preliminary findings were reported descriptively overall and per randomised group. For categorical outcomes, the proportion of patients having adverse events and being satisfied with care (to a high or very high degree) were reported with 95%CI. For continuous outcomes, the mean pain intensity and disability were reported with standard deviation (SD).

### Progression criteria

Progression criteria (Table 1) were defined to identify where modifications to the trial processes may be required to ensure feasibility or where progression to a large-scale trial should not be considered [32]. The research team identified factors that would impact feasibility of a large-scale RCT and identified indicators to inform the progression criteria. Whether feasibility could be achieved through more funding or resources (e.g., recruitment numbers, follow-up) was considered when determining the progression criteria. The progression criteria were not designed to be absolute cut-points, but rather to ensure appropriate consideration was given to identified issues before proceeding to a large-scale trial.

**Table 1:** Progression criteria to identify feasibility of progressing to a large-scale RCT

Feasibility component	Proceed (no major problems identified)	Proceed with modification (problems identified can be addressed)	Do not proceed (problems identified can’t be addressed)
Chiropractor recruitment	≥ 20% chiropractors screened meet inclusion criteria; ≥ 75% chiropractors meeting inclusion criteria consent; ≥ 10 chiropractors recruited	≥ 20% chiropractors screened meet inclusion criteria; 50-<75% chiropractors meeting inclusion criteria consent; 5–9 chiropractors recruited	< 20% chiropractors screened meet inclusion criteria; < 50% chiropractors meeting inclusion criteria consent; < 5 chiropractors recruited
Patient recruitment	≥ 20% patients screened meet inclusion criteria; ≥ 75% patients meeting inclusion criteria consent; ≥ 50 patients recruited	≥ 20% patients screened meet inclusion criteria; 50–< 75% patients meeting inclusion criteria consent; ≥ 20 patients recruited	< 20% patients screened meet inclusion criteria; < 50% patients meeting inclusion criteria consent; < 20 patients recruited
Compliance with randomisation	≥ 85% compliance with randomisation, with equal cross-over occurring in both groups	≥ 70% compliance with randomisation, with cross-over occurring in both groups	< 70% compliance with randomisation or all cross-over occurring in one group
Compliance with study processes	≥ 80% compliance with all study processes (chiropractor recruitment of at least 1 participant, chiropractor and patient questionnaire completion)	≥ 50% compliance with all study processes (chiropractor recruitment of at least 1 participant, chiropractor and patient questionnaire completion)	< 50% compliance with all study processes (chiropractor recruitment of at least 1 participant, chiropractor and patient questionnaire completion)
Acceptability of trial	No major acceptability issues identified	Acceptability issues identified but able to be addressed	Acceptability issues identified and not able to be addressed
Preliminary findings	Trends in patient outcomes, adverse events, and satisfaction with care are consistent with published research with no differences between groups	Trends in patient outcomes, adverse events, and satisfaction with care are consistent with published research with small differences between groups	Trends in patient outcomes, adverse events, and satisfaction with care are inconsistent with published research or show large differences between groups

## Results

### Chiropractor and patient recruitment (aim 1)

**Fig. 1:**
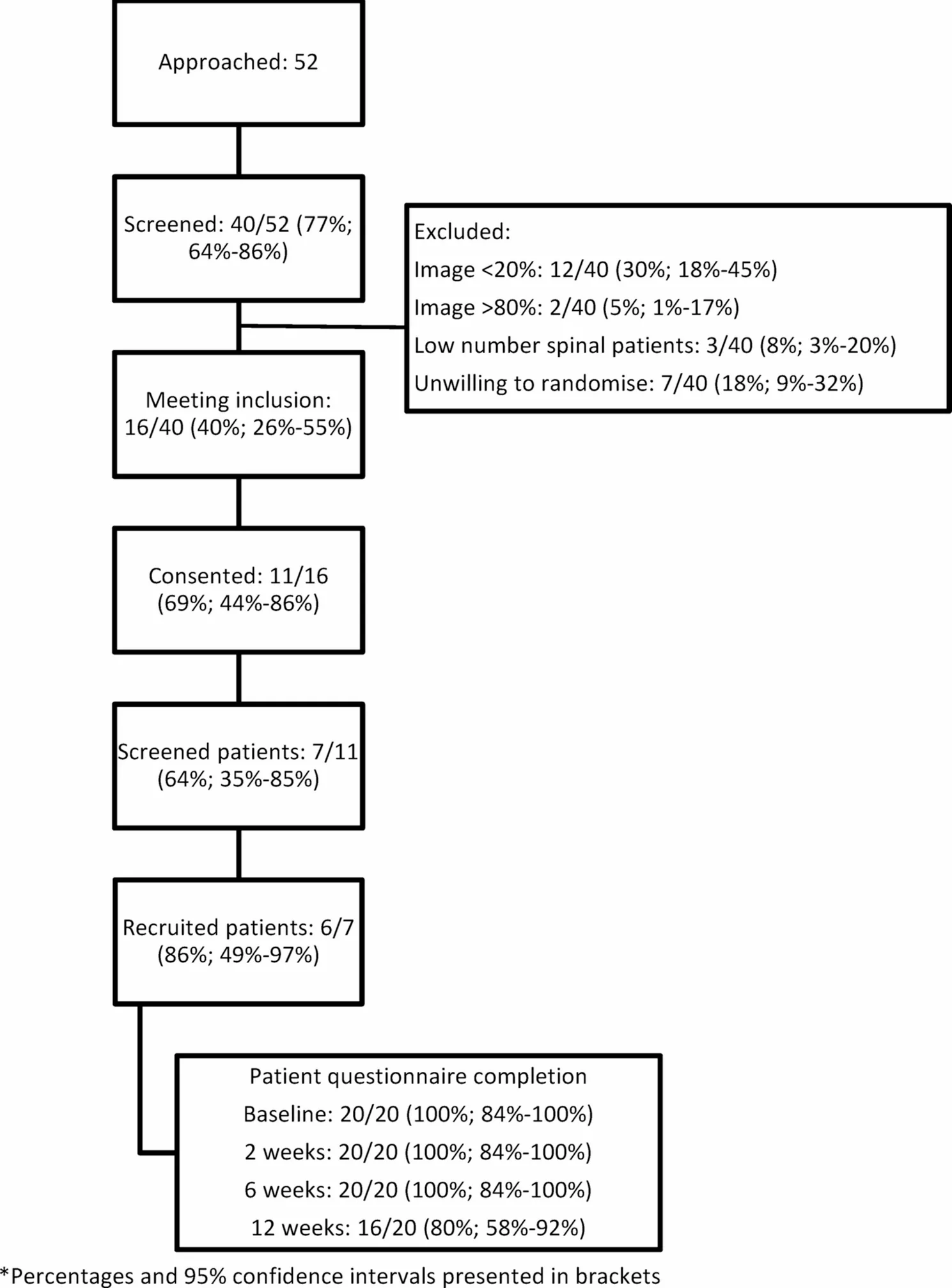
Proportions of chiropractors meeting each stage of recruitment and study processes*

Recruitment of chiropractors met the ‘Proceed’ or ‘Proceed with modification’ progression criteria. Of 40 chiropractors screened for inclusion in the study, 16/40 (40%; 95%CI 26–55) met inclusion criteria and 11/16 (69%; 95%CI 44–86) consented to participate. Of those excluded, 30% reported performing diagnostic imaging in less than 20% of cases and 18% were unwilling to randomise patients to receive or not receive imaging (Fig. 1). While all participating chiropractors met inclusion criteria for imaging referral on initial screening, three participants subsequently reported referring 10–20% of new patients for imaging on the baseline questionnaire.

Recruited chiropractors were mostly male (8/11, 73%) and nearly all completed their chiropractic education at a single institution (10/11, 91%). Broad spread was seen in years in clinical practice, location of radiology referral (in-house or to an external radiology centre), reported frequency of previous radiographic referral, and beliefs regarding imaging use (Table 2).

**Table 2:** Characteristics of included chiropractors, stratified by level of patient recruitment in the study*

	Overall	High recruiters	Low recruiters	No recruitment
Age (mean, SD)	39.7 (16)	47.5 (4)	35.7 (9)	44.1 (11)
Sex (n/N, % Female)	3/11 (27)	1/3 (33)	0/3 (0)	2/5 (40)
Years in practice (mean, SD, range)	19 (9, 4–31)	25 (4, 11–31)	11 (7, 4–18)	20 (10, 21–29)
Chiropractic education (n/N, %)				
Macquarie University/Sydney College	10/11 (91)	2/3 (67)	3/3 (100)	5/5 (100)
RMIT University	1/11 (9)	1/3 (33)	0/3 (0)	0/5 (0)
Hours in clinical practice/week (mean, SD, range)	32 (9, 12–40)	35 (5, 30–38)	37 (4, 32–40)	26 (11, 12–40)
Frequency of radiography referral (n/N, %)				
10%-20% of new patients	3/11 (27)	0/3 (0)	1/3 (33)	2/5 (40)
21%-50% of new patients	4/11 (36)	2/3 (67)	1/3 (33)	1/5 (20)
51% to 80% of new patients	4/11 (36)	1/3 (33)	1/3 (33)	2/5 (40)
Location of radiography referral (n/N, %)				
Own radiology facilities	4/11 (36)	2/3 (67)	0/3 (0)	2/5 (40)
Other chiropractor radiology facilities	1/11 (9)	0/3 (0)	1/3 (33)	0/5 (0)
Medical radiology facilities	6/11 (55)	1/3 (33)	2/3 (67)	3/5 (60)
Reasons for new patient radiography referral (n/N, %)				
Serious pathology	9/11 (82)	3/3 (100)	3/3 (100)	3/5 (60)
Fracture	11/11 (100)	3/3 (100)	3/3 (100)	5/5 (100)
Screening for contraindications for spinal manipulation	2/11 (18)	0/3 (0)	1/3 (33)	1/5 (20)
Benign spinal pathology	11/11 (100)	3/3 (100)	3/3 (100)	5/5 (100)
Biomechanical analysis	3/11 (27)	3/3 (100)	0/3 (0)	0/5 (0)
At patient request	3/11 (27)	0/3 (0)	1/3 (33)	2/5 (40)
Imaging use beliefs (n/N, % Agree)				
Useful prior to initiating spinal manipulative therapy	5/11 (56)	2/3 (67)	1/3 (33)	2/5 (40)
Useful in the workup of patients with acute spinal pain	4/11 (44)	1/3 (33)	2/3 (67)	1/5 (20)
Likely to order radiographs for acute back or neck pain	4/11 (44)	1/3 (33)	1/3 (33)	2/5 (40)

*Low recruiters defined as chiropractors who recruited 1–3 patients into the study; high recruiters recruited more than 3 patients

**Fig. 2:**
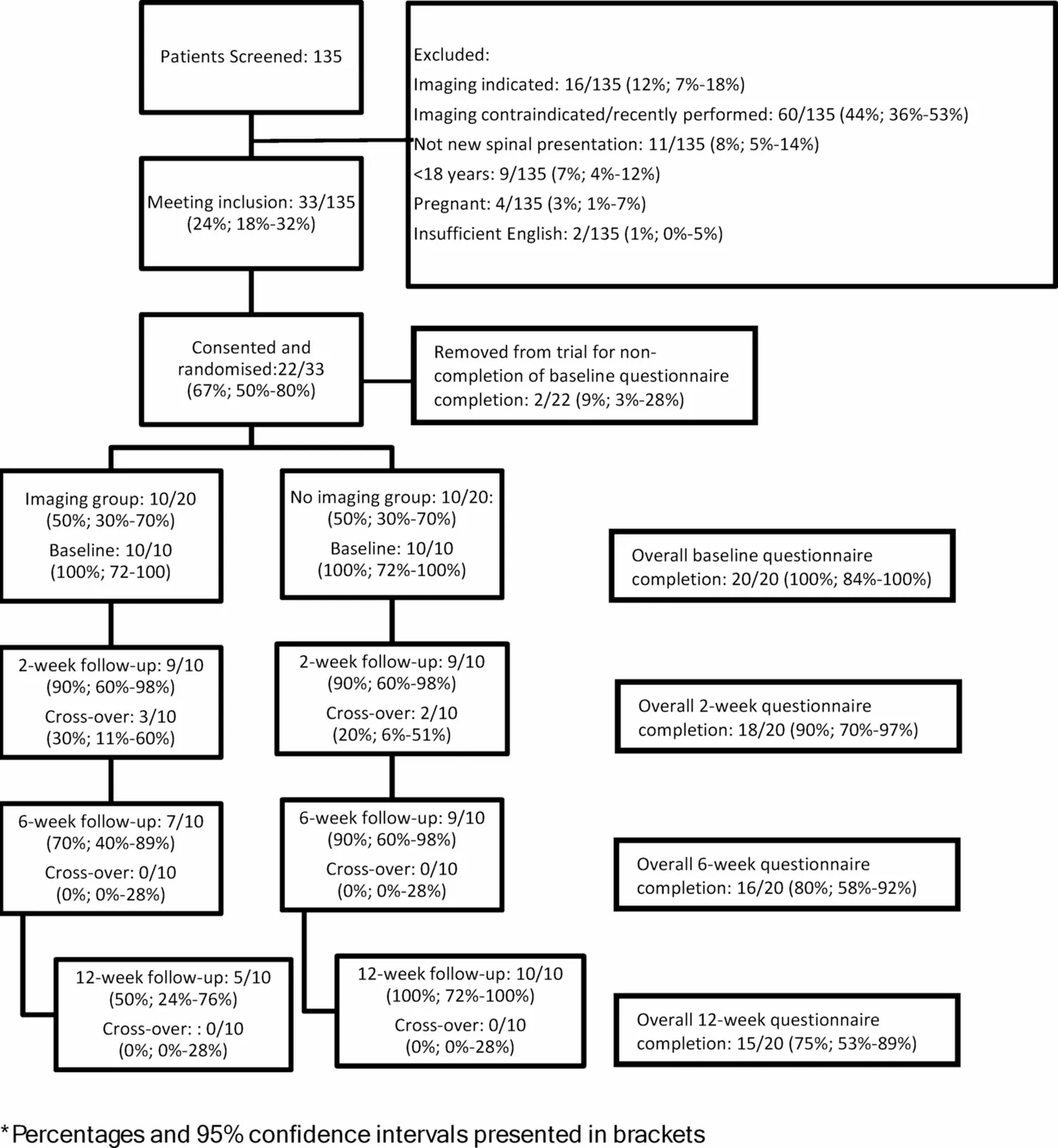
Study flow chart*

Recruitment of patients met the ‘Proceed with modification’ progression criteria. Participating chiropractors screened 135 patients (mean: 12; standard deviation (SD): 17; range: 0–56) across the two study periods, with 33/135 (24%; 95%CI 18–32) meeting inclusion criteria and 22/33 (67%; 95%CI 50–80) consenting to participate. Of those excluded, 44% were either contraindicated for imaging or had received recent imaging and 12% were strongly indicated for imaging (Fig. 2). Two consenting participants did not complete the baseline questionnaire or participate in the trial and were excluded from the analysis.

Included patients had varied educational background and health insurance status but tended to be young (mean age 36 years) and have an Anglo-European cultural background (85%). Back and neck pain were present in 85% and 65% of participants respectively, with moderate mean pain intensity and low disability. Approximately half of participants (55%) believed that radiographs are necessary to get the best medical care for back or neck pain (Table 3).

**Table 3:** Characteristics of included patients, stratified by randomisation group

	Overall (*N* = 20)	Intervention (*N* = 10)	Control (*N* = 10)
Age (mean, SD)	36 (17)	33 (19)	38 (15)
Sex (n/N, % Female)	12/20 (60)	7/10 (70)	5/10 (50)
Educational background (n/N, %)			
Secondary education	5/20 (25)	2/10 (20)	3/10 (30)
Vocational training	7/20 (35)	4/10 (40)	3/10 (30)
Tertiary education	8/20 (40)	4/10 (40)	4/10 (40)
Cultural background (n/N, %)			
Anglo-European	17/20 (85)	8/10 (80)	9/10 (90)
Asian	2/20 (10)	1/10 (10)	1/10 (10)
African	1/20 (5)	1/10 (10)	0/10 (0)
Health insurance status (n/N, %)			
Private insurance	14/20 (70)	7/10 (70)	7/10 (70)
Medicare only	6/20 (30)	3/10 (30)	3/10 (30)
Back pain presentation (n/N, %)	17/20 (85)	9/10 (90)	8/10 (80)
Previous back pain (n/N, %)	14/17 (82)	7/9 (78)	7/8 (88)
Previous chiropractic treatment (n/N, %)	11/17 (65)	5/9 (56)	6/8 (75)
Back pain intensity 0–10 (mean, SD)	5 (1)	5 (2)	5 (1)
Back pain disability 0–24 (mean SD)	6 (4)	4 (3)	9 (4)
Neck pain presentation (n/N, %)	13/20 (65)	6/10 (60)	7/10 (70)
Previous neck pain (n/N, %)	10/13 (77)	5/6 (83)	5/7 (71)
Previous chiropractic treatment (n/N, %)	7/13 (54)	3/6 (50)	4/7 (57)
Neck pain intensity 0–10 (mean, SD)	4 (2)	4 (2)	4 (2)
Neck pain disability 0–50 (mean, SD)	8 (2)	8 (2)	9 (3)
Previous imaging for spinal pain (n/N, %)	6/20 (30)	3/10 (30)	3/10 (30)
Radiographs	5/6 (83)	2/3 (66)	3/3 (100)
CT	2/6 (33)	1/3 (33)	1/3 (33)
Imaging use beliefs (n/N, % Agree)			
Radiographs necessary for best medical care	11/20 (55)	5/10 (50)	6/10 (60)
Everyone should have spinal radiographs	6/20 (30)	3/10 (30)	3/10 (30)

### Compliance with the randomisation schedule (aim 2)

Compliance with randomisation met the ‘Proceed with modification’ progression criteria. Of the 20 participants who consented to participate and completed the baseline questionnaire, 10 were randomised to the intervention group and 10 to the control group. Participants in the intervention group tended to be younger (mean age 33 years compared to 38 years), female (70% compared to 50%), and have less back pain disability (mean RMDQ 4/24 compared to 9/24).

Cross-over between groups occurred for 3/10 participants randomised to the intervention group (30%; 95%CI 11–60) and 2/10 participants randomised to the control group (20%; 95%CI 6–51) within the first-two weeks. All decisions to either obtain or not obtain radiographs inconsistent with the randomisation schedule were made in consultation with the treating chiropractor due to changes in the patients’ clinical presentation. The two patients randomised to the no-imaging group who subsequently obtained imaging presented with worsening clinical symptoms within the first 2-weeks. One received spinal radiographs and the other a lumbar MRI. The three patients randomised to the imaging group who did not receive imaging had early and meaningful improvements in their clinical symptoms.

### Compliance with study processes and acceptability of the trial (aim 3)

#### Patient screening and recruitment

Patient screening and recruitment met the ‘Proceed with modification’ progression criteria. On average, the participating chiropractors screened 12 patients (SD: 17; Range: 0–56) and recruited two patients (SD: 2.9; Range: 0–9). Seven of the 11 chiropractors (64%; 95%CI 35–85) screened at least one patient and of these, 6/7 (86%; 95%CI 49–97) recruited at least one patient (Fig. 1). Three of the six chiropractors who recruited participants were categorised as high recruiters, screening 33 patients (SD: 20; Range: 21–56) and recruiting six patients (SD: 3; Range: 4–9), on average. In contrast, low recruiters (*n* = 3) screened, on average, 11 patients (SD: 7; Range 7–19) and recruited one patient (SD: 1; Range 1–2). Higher recruiters tended to have been in clinical practice for longer (mean years 25 compared to 11) and have their own radiographic facilities (67% compared to 0%) (Table 2).

The COVID-19 pandemic was identified as one of the largest challenges to screening and recruitment due to decreased patient flow through the clinic and other business stresses making them less likely to think about the trial.

“Okay, it’s been very hectic and there’s been very few new patients due to COVID at this stage” *(ID39*,* male*,* 31 years in practice*,* refer 10–20% of new patients for spinal radiographs)*.

While the screening form was considered concise and easy to use, chiropractors reported that they felt it would take more time, which made them less likely to complete patient screening.

“Aside from our paperwork and everything, there was all that extra paperwork to do, even if there wasn’t that much” *(ID9*,* male*,* 12 years in practice*,* refer 51–80% of new patients for spinal radiographs)*.

The time needed to explain the trial to the patient and the potential workload for the patient were also viewed as barriers to recruitment.

“There were probably more people I could have enrolled but didn’t just because it was quicker for me to not do it” *(ID24*,* male*,* 4 years in practice*,* refer 10–20% of new patients for spinal radiographs)*.

“Patients are on the go and if they can’t benefit from the study, then it’s another exercise that’s consuming that everyday life, and so there wasn’t a great incentive for them to be fulfilling the study” *(ID47*,* male*,* 14 years in practice*,* refer 51–80% of new patients for spinal radiographs)*.

Chiropractors reported significant challenges in identifying people who met the inclusion criteria and then explaining the trial to them. Chiropractors felt their clinical skills were sufficient to determine whether a person needed imaging or not, and that the diagnostic uncertainty required to recruit someone into the trial was not present in many cases.

“there’s only a very limited sort of subset of patients that are going to fit this criteria, right, because I’m not a chiropractor that just x-rays everybody. And I’m certainly not a chiropractor that doesn’t x-ray anyone, right. So, for me, there are certain indications for x-ray so it’s been a little bit challenging” (*ID36*,* male*,* 29 years in practice*,* refer 21–50% of new patients for spinal radiographs)*.

When chiropractors identified someone who met inclusion criteria, some found explaining the uncertainty around the need for diagnostic imaging challenging. They believed patients may be concerned that they weren’t receiving appropriate management and that this may impact their continuation with care.

“most, if not all, patients were I guess anxious about potentially being randomised into the group that don’t have the x-ray….and they’d rather have the x-ray taken to be, you know, to be safer or to share that sense of assurance that everything is okay” *(ID47*,* male*,* 14 years in practice*,* refer 51–80% of new patients for spinal radiographs)*.

“I didn’t really want to coax patients into doing the study, was just fear of losing them” *(ID9*,* male*,* 12 years in practice*,* refer 51–80% of new patients for spinal radiographs)*.

Some chiropractors thought that increased training with case scenarios may help their ability to explain the trial to patients. Others felt that it was easier to explain the trial when there was good rapport with the patient and when they highlighted the importance of being involved in the trial.

“He was an existing the patient, so I knew him well. So, it was easier to approach him with the study, I guess. I think that might have been different, having rapport with the patient first, before starting the recruitment process.” *(ID9*,* male*,* 12 years in practice*,* refer 51–80% of new patients for spinal radiographs)*.

#### Completion of patient questionnaires

Completion of follow-up questionnaires by both patients and chiropractors broadly met the ‘Proceed’ progression criteria. All participating chiropractors completed the questionnaires for each patient they enrolled in the study at baseline, 2-weeks, and 6-weeks. At 12-weeks 16/20 (80%; 95%CI 58–92) questionnaires were completed (Fig. 1). Chiropractors reported finding the follow-up questionnaires easy to complete. Some chiropractors felt they needed to add further information including whether they were still treating the patient and reasons for imaging use:

“I wanted to keep writing more info to give you like why did I take this x-ray? What did I do with it?” (*ID36*,* male*,* 29 years in practice*,* refer 21–50% of new patients for spinal radiographs)*.

Overall completion of questionnaires by participating patients decreased from 90% at 2-weeks (95%CI 70–97) to 75% at 12-weeks (95%CI 53–89). Questionnaire completion was performed better in the control group at 6-weeks and 12-weeks (Fig. 2).

#### Acceptability of the trial

Acceptability of the trial met the ‘Proceed with modification’ progression criteria. Participating chiropractors thought the trial was important to improve clinical decision-making due to the variability in imaging use between chiropractors.

“a study which I thought was important for our profession in terms of making better diagnosis, so we could provide patients with better outcomes.” (*ID5*,* female*,* 24 years in clinical practice*,* refer 51–80% of new patients for spinal radiographs)*.

However, there was concern that acknowledging diagnostic uncertainty to patients challenged their professional identity and that being involved in the trial may result in imaging referral decisions out of alignment with their clinical expertise.

“The hardest thing I’ve found is it sort of challenged my expertise, basically because you’re saying to the patient I can send you for these x-rays, but they’re not necessary” *(ID24*,* male*,* 4 years in practice*,* refer 10–20% of new patients for spinal radiographs)*.

“And I held off on it [because of the trial], I thought, but in the back of my head I knew I should have imaged her” (*ID5*,* female*,* 24 years in clinical practice*,* refer 51–80% of new patients for spinal radiographs)*.

Some chiropractors were concerned that if patients were randomised to the no-imaging group they may miss important clinical indicators that could affect their treatment. However, only one of the 10 control patients did not receive spinal manipulation as originally intended (10%; 95%CI 2–40).

### Preliminary findings (aim 4)

Preliminary findings broadly met the ‘Proceed with modification’ progression criteria. Pain intensity and disability of the back and neck tended to decrease over time in both groups (Table 4). Satisfaction with care was generally high, ranging from 83% of participants with high or very-high satisfaction with care at 2-weeks (15/18; 61%-94%) to 94% of participants at 6-weeks (15/16; 72%-99%).

**Table 4:** Patient-reported outcomes, stratified by randomisation group

	Overall	Intervention	Control
Low back pain intensity (0–10; mean, SD)			
2 weeks (*N* = 15 overall; 8 intervention; 7 control)	5.1 (2.1)	4.4 (2.4)	6.0 (1.4)
6 weeks (*N* = 13 overall; 6 intervention; 7 control)	3.3 (1.7)	2.7 (1.4)	3.9 (1.9)
12 weeks (*N* = 11 overall; 4 intervention; 7 control)	3.8 (3.0)	5.0 (4.2)	3.1 (2.2)
Low back disability (0–24; mean, SD)			
2 weeks (*N* = 15 overall; 8 intervention; 7 control)	5.3 (5.4)	4.5 (5.6)	6.1 (5.5)
6 weeks (*N* = 13 overall; 6 intervention; 7 control)	2.8 (2.5)	1.8 (2.3)	3.7 (2.6)
12 weeks (*N* = 11 overall; 4 intervention; 7 control)	1.6 (2.6)	1.3 (1.0)	1.9 (3.2)
Neck pain intensity (0–10; mean, SD)			
2 weeks *n* = 13 (*n* = 6 intervention; *n* = 7 control)	4.5 (2.5)	4.8 (2.9)	4.3 (2.2)
6 weeks *n* = 12 (*n* = 5 intervention; *n* = 7 control)	3.2 (1.8)	2.8 (1.1)	3.4 (2.2)
12 weeks *n* = 10 (*n* = 4 intervention; *n* = 6 control)	3.5 (3.1)	4.8 (3.9)	2.7 (2.5)
Neck disability (0–50; mean, SD)			
2 weeks *n* = 13 (*n* = 6 intervention; *n* = 7 control)	5.4 (3.9)	6.0 (4.6)	4.9 (3.5)
6 weeks *n* = 12 (*n* = 5 intervention; *n* = 7 control)	5.9 (2.9)	5.6 (1.3)	6.1 (3.8)
12 weeks *n* = 11 (*n* = 4 intervention; *n* = 7 control)	4.5 (3.8)	3.8 (3.8)	4.9 (4.0)
Satisfaction with care (proportion, %, 95%CI)			
2 weeks	15/18 (83%; 61%-94%)	8/9 (89%; 57%-98%)	7/9 (78%; 45%-94%)
6 weeks	15/16 (94%; 72%-99%)	7/7 (100%; 65%-100%)	8/9 (89%; 57%-98%)
12 weeks	13/15 (87%; 62%-96%)	5/5 (100%; 57%-100%)	8/10 (80%; 49%-94%)
Adverse events (proportion, %, 95%CI)	4/20 (20%; 8%-42%)	1/10 (10%; 2%-40%)	3/10 (30%, 11%-60%)

Adverse events were infrequent (4/20 participants; 20%; 95%CI 8–42), mild, and transient and occurred in both groups (imaging group: 1/10, 10%, 95%CI 2–40; no-imaging group: 3/10, 30%, 95%CI 11–60). Adverse events included increased muscle soreness and reaggravation of presenting pain.

## Discussion

This study piloted the study design and processes planned for a large-scale randomised controlled trial to test whether referring for spinal radiographs in patients receiving spinal manipulative therapy affects patient outcomes or changes the frequency or degree of adverse events. Feasibility of many aspects of a full-scale effectiveness study were demonstrated, while challenges and potential solutions were also identified. No ‘Do not proceed’ progression criteria were identified.

### Chiropractor recruitment

We met our feasibility target of 10 chiropractors (11 chiropractors recruited) and these included a broad range of demographic and clinical practice characteristics. To meet this target, 52 chiropractors were initially approached. More than three-quarters of those approached requested more information about the study and were screened for inclusion criteria, a higher proportion than seen in clinician recruitment in other settings [33, 34]. However, of those screened, 60% did not meet inclusion criteria, because they either reported using imaging too frequently (5%) or infrequently (30%) or were not willing to randomise patients to receive imaging (18%). While these inclusion criteria could be broadened to facilitate chiropractor recruitment, it is important that the chiropractors will be engaged with the study and willing and able to recruit patients [35, 36]. For example, despite reporting higher than 20% imaging rates at the time of screening, three of the participating chiropractors (27%) subsequently reported referring less than 20% of new patients for radiographic imaging on the baseline questionnaire. These chiropractors recruited few patients into the trial, and more strict assessment of this inclusion criteria may be needed in future RCTs. Of those chiropractors who met inclusion criteria, 69% consented to participate, a similar proportion to clinician recruitment in other settings [33]. Participating chiropractors thought the trial was important for the profession and likely to provide evidence to improve clinical practice and outcomes.

### Patient screening and recruitment

Patient recruitment did not meet the feasibility target of 50 patients (five patients per clinician), with only 22 patients consenting and 20 participating in the trial. Chiropractors identified that patient recruitment was impacted by the COVID-19 pandemic due to interruption to the study processes between the two study periods, fewer new patients attending clinics, and other business concerns they needed to focus on. Patient recruitment was variable between clinicians, ranging from recruiting zero patients to nine patients, and was likely impacted by a variety of factors. Recruitment concerns included difficulty in adding the screening process into a clinical consult, uncertainty around who should be recruited into the study, and uncertainty on how to explain the study to patients.

Recruitment in clinical practice is recognised to be challenging, with variable recruitment between clinicians commonly observed [37]. Recruitment tends to be more successful when strategies can be largely managed by the research team, thus reducing clinician burden [36]. In this study, clinicians were needed to initially identify patients who met inclusion criteria after their clinical assessment. However, it may be possible for the research team to take on more responsibility for explaining the study to the patients to reduce burden on the clinicians. Having the research team explain the study could also decrease the chiropractors’ concern that explaining the uncertainty around imaging decisions to the patient may lessen their professional authority, thus decreasing another barrier to patient recruitment. It may additionally reduce the risk of coercion and undue influence from practitioners, provide more consistent and standardised information delivery, and provide a clearer distinction between the role of the practitioner and researchers for patients.

While all recruited chiropractors were initially willing to randomise patients to receive or not receive spinal radiographs, in practice this did not always occur. Even in patients without any indicators of serious pathology, chiropractors reported being unwilling to recruit patients into the study when they thought that imaging was either needed or not needed and therefore did not wish to randomise the patient. While we anticipated this concern and provided detailed information on selecting patients for inclusion to try and mitigate the issue, qualitative feedback indicated that the chiropractors’ underlying beliefs about the use of imaging tended to impact their decision-making. Typically, low or no recruiters were chiropractors who either reported low or high use of spinal radiographs prior to the study, which aligned with their beliefs on the importance of spinal radiographs in clinical practice.

The recruited population should reflect the population of interest to ensure generalisability of results. Screening of consecutive patients is important to limit selection bias [36]; however, this is challenging in clinical practice where other clinical demands may impact patient screening. In this study, 135 patients were screened, and while it is unlikely that this was all consecutive new patients presenting to the clinics, chiropractors were recording those that both met and did not meet inclusion criteria, with approximately three-quarters excluded on screening. Included patients were, on average, young compared to populations presenting for chiropractic care in the literature [4, 38], with only one patient over the age of 65. This young age demographic may reflect the chiropractors’ decision of whom to include in the study, as older age is a potential indicator for imaging referral, particularly if fracture is suspected [5]. Recruiting older patients into trials can be challenging [39]. Greater consideration may be needed to increase older patient recruitment, including addressing patient or clinician concerns about perceived increased need for imaging. Importantly, recruited patients had similar beliefs on the importance of imaging as the general population [24], indicating that recruitment didn’t bias towards or away from patients with stronger beliefs about the importance of imaging.

### Compliance with the randomisation schedule and study processes

Cross-over occurred in 20% of those randomised to not receive imaging, and in 30% of those randomised to receive imaging. All cross-over decisions were made with the treating chiropractor, who determined that radiography was either indicated or not indicated at a later time-point. Indications or non-indications for imaging are not always apparent at the first consultation, and review of clinical decision-making is consistent with imaging referral guidelines [5]. However, cross-over may also be related to underlying patient or clinician beliefs and strategies to limit cross-over between groups would be needed in a large-scale trial to minimise associated bias. Strategies could include increased training for the chiropractors on the inclusion criteria and identifying indicators or non-indicators for imaging, obtaining imaging in a timely fashion for those randomised to the radiography group, provision of a script with clear explanations for the patient regarding uncertainty around the need for imaging and the importance of adherence to the randomised allocation, and measurement of satisfaction with randomisation assignment to assess expectation bias.

Almost complete follow-up was performed by the chiropractors, whereas patient follow-up ranged from 90% at 2-weeks to 75% at 12-weeks. This pilot study was not resourced to provide sufficient research assistance to facilitate high follow-up rates as seen in other trials [40, 41]. While chiropractors were reimbursed a small amount per patient for their involvement, no patient reimbursement was provided, a limitation highlighted by the chiropractors in qualitative feedback. A large-scale trial should consider the need for appropriate research assistance and participant reimbursement to ensure optimal data collection.

### Preliminary findings

Preliminary findings were presented to assess for large differences in adverse events, pain outcomes, or satisfaction with care between groups that may impact the safety or need for a large-scale trial. It must be noted that this pilot trial was not powered to detect clinically significant differences between groups, and for this reason trends in outcome findings over time were presented rather than between-group effects. Differences were typically small (e.g., differences between 0.5 and 1 points on a 10-point scale) and should not be interpreted as differences in effect between groups.

Reported adverse events were infrequent, transient, and mild, occurring in approximately 20% of participants as consistent with adverse events observed in other trials of chiropractic care [29]. Chiropractors report using radiographs to determine the appropriateness of applying force to the spine and whether spinal manipulation can be applied [23, 24], despite evidence of the low sensitivity of radiography for detecting important contraindications [14, 15]. This concern was also raised by participating chiropractors; however, they did not report that lack of radiography directly impacted their decision to perform spinal manipulation for patients in the study. Only one participant (randomised to the no radiography group) did not receive spinal manipulation as originally intended, but it is not known whether spinal manipulation would have been used if radiography had been performed or whether other clinical changes impacted the decision. The inconsistency between chiropractic clinical practice and evidence-based recommendations regarding the use of radiography further highlights the need for a large-scale RCT.

### Limitations

Limitations included that the study had limited generalisability of chiropractors who participated, it probably lacked consecutive patient recruitment, it was underpowered to detect clinically significant differences between groups, and patient participants were younger than the general chiropractic patient population. Feasibility targets for patient recruitment were not met. While patient recruitment was likely negatively impacted by interruptions due to the COVID-19 pandemic, strategies to increase recruitment of consecutive patients should be considered for a large-scale trial. While satisfaction with care was high in both groups, we did not collect data on whether the trial processes directly impacted patient compliance with treatment.

## Conclusion

Chiropractor recruitment, participant follow-up, and preliminary findings met progression criteria to conduct a large-scale RCT to test the effect of referring for spinal radiography prior to performing spinal manipulative therapy in chiropractic practice. However, suitable strategies would be required to ensure feasibility of patient recruitment and reduce cross-over between groups. The generalisability of results in a large-scale RCT may be limited by the recruitment of chiropractors willing to randomise patients to receive radiographs and the strict patient inclusion criteria.

## Supplementary Information

Below is the link to the electronic supplementary material.


Supplementary Material 1


## Data Availability

The datasets used and/or analysed during the current study are available from the corresponding author on reasonable request.

## References

[1] Young KJ. Evaluation of publicly available documents to trace chiropractic technique systems that advocate radiography for subluxation analysis: a proposed genealogy. J Chiropr Humanit. 2014;21(1):1–24. 25431540 10.1016/j.echu.2014.09.001 PMC4245702

[2] Bolton SP. X-ray dispossessed-expedience versus standards? Chiropr J Aust. 2004;34(1):23–9.

[3] Adams J, Lauche R, Peng W, Steel A, Moore C, Amorin-Woods LG, et al. A workforce survey of Australian chiropractic: the profile and practice features of a nationally representative sample of 2,005 chiropractors. BMC Complement Altern Med. 2017;17(1):14. 28056964 10.1186/s12906-016-1542-x PMC5217252

[4] French SD, Charity MJ, Forsdike K, Gunn JM, Polus BI, Walker BF, et al. Chiropractic Observation and Analysis Study (COAST): providing an understanding of current chiropractic practice. Med J Aust. 2013;199(10):687–91. 24237100 10.5694/mja12.11851

[5] Jenkins HJ, Downie AS, Moore CS, French SD. Current evidence for spinal X-ray use in the chiropractic profession: a narrative review. Chiropr Man Ther. 2018;26(1):48. 10.1186/s12998-018-0217-8 PMC6247638 30479744

[6] Corso M, Cancelliere C, Mior S, Kumar V, Smith A, Côté P. The clinical utility of routine spinal radiographs by chiropractors: a rapid review of the literature. Chiropr Man Ther. 2020;28(1):33. 10.1186/s12998-020-00323-8 PMC7346665 32641135

[7] Bussieres A, Taylor J, Peterson C. Diagnostic imaging practice guidelines for musculoskeletal complaints in adults—an evidenced-based approach—part 3: spinal disorders. J Manip Physiol Ther. 2008;31:33–88. 10.1016/j.jmpt.2007.11.003 18308153

[8] Oakley PA, Cuttler JM, Harrison DE. X-ray imaging is essential for contemporary chiropractic and manual therapy spinal rehabilitation: radiography increases benefits and reduces risks. Dose-Response. 2018;16(2):1559325818781437. 29977177 10.1177/1559325818781437 PMC6024283

[9] Kawchuk G, Goertz C, Axén I, Descarreaux M, French S, Haas M, et al. Letter to the Editor Re: Oakley PA, Cuttler JM, Harrison DE. X-ray imaging is essential for contemporary chiropractic and manual therapy spinal rehabilitation: radiography increases benefits and reduces risks. Dose Response. 2018;16(2):1559325818811521. 10.1177/1559325818811521 PMC6311565 30627066

[10] EPIC WFC ECU Congress. Academic programme; 2019. Available from: https://www.epic2019.net/wp-content/uploads/sites/62/2019/02/EPIC2019-Draft-Academic-Programme-2019_02_13.pdf.

[11] Jenkins HJ. Awareness of radiographic guidelines for low back pain: a survey of Australian chiropractors. Chiropr Man Ther. 2016;24(1):1–9. 10.1186/s12998-016-0118-7 PMC5051064 27713818

[12] De Carvalho D, Bussières A, French SD, Wade D, Brake-Patten D, O’Keefe L, et al. Knowledge of and adherence to radiographic guidelines for low back pain: a survey of chiropractors in Newfoundland and Labrador, Canada. Chiropr Man Ther. 2021;29(1):1–10. 10.1186/s12998-020-00361-2 PMC7812732 33461555

[13] Bussieres AE, Patey AM, Francis JJ, Sales AE, Grimshaw JM, Brouwers M, et al. Identifying factors likely to influence compliance with diagnostic imaging guideline recommendations for spine disorders among chiropractors in North America: a focus group study using the Theoretical Domains Framework. Implement Sci. 2012;7:1–11. 10.1186/1748-5908-7-82 PMC3444898 22938135

[14] Joseph SY, Krishna NG, Fox MG, Blankenbaker DG, Frick MA, Jawetz ST, et al. ACR appropriateness criteria^®^ osteoporosis and bone mineral density: 2022 update. 2022;19(11):S417–32. 10.1016/j.jacr.2022.09.007 36436967

[15] American College of Radiologists. ACR appropriateness criteria; 2013. Available from: http://www.acr.org/Quality-Safety/Appropriateness-Criteria.

[16] Flynn TW, Smith B, Chou R. Appropriate use of diagnostic imaging in low back pain: a reminder that unnecessary imaging may do as much harm as good. J Orthop Sports Phys Ther. 2011;41(11):838–46. 10.2519/jospt.2011.3618 21642763

[17] Hall AM, Aubrey-Bassler K, Thorne B, Maher CG. Do not routinely offer imaging for uncomplicated low back pain. BMJ. 2021;372:n291. 10.1136/bmj.n291 PMC8023332 33579691

[18] Webster B, Bauer AS, Choi Y, Cifuentes M, Pransky G. Iatrogenic consequences of early MRI in acute work-related disabling low back pain. Spine. 2013;38(22):1939–46. 23883826 10.1097/BRS.0b013e3182a42eb6 PMC4235393

[19] Chou R, Fu R, Carrino J, Deyo R. Imaging strategies for low-back pain: systematic review and meta-analysis. Lancet. 2009;373:463–72. 19200918 10.1016/S0140-6736(09)60172-0

[20] Djais N, Kalim H. The role of lumbar spine radiography in the outcomes of patients with simple acute low back pain. APLAR J Rheumatol. 2005;8(1):45–50.

[21] Kendrick D, Fielding K, Bentley E, Kerslake R, Miller P, Pringle M. Radiography of the lumbar spine in primary care patients with low back pain: randomised controlled trial. BMJ. 2001;322(7283):400–5. 11179160 10.1136/bmj.322.7283.400 PMC26570

[22] Jenkins HJ, Kongsted A, French SD, Jensen TS, Doktor K, Hartvigsen J, et al. What are the effects of diagnostic imaging on clinical outcomes in patients with low back pain presenting for chiropractic care: a matched observational study. Chiropr Man Ther. 2021;29(1):46. 10.1186/s12998-021-00403-3 PMC8611826 34814923

[23] Searant I, Brown BT, Jenkins HJ. Chiropractors’ perceptions on the use of spinal radiographs in clinical practice: a qualitative study. Chiropr Man Ther. 2024;32(1):23. 10.1186/s12998-024-00547-y PMC11193277 38909258

[24] Jenkins HJ, Hancock MJ, Maher CG, French SD, Magnussen JS. Understanding patient beliefs regarding the use of imaging in the management of low back pain. Eur J Pain. 2016;20(4):573–80. 26282178 10.1002/ejp.764

[25] Jenkins HJ, Kongsted A, French SD, Jensen TS, Doktor K, Hartvigsen J, et al. Patients with low back pain presenting for chiropractic care who want diagnostic imaging are more likely to receive referral for imaging: a cross-sectional study. Chiropr Man Ther. 2022;30(1):16. 10.1186/s12998-022-00425-5 PMC8978373 35379281

[26] Eldridge SM, Chan CL, Campbell MJ, Bond CM, Hopewell S, Thabane L, et al. CONSORT 2010 statement: extension to randomised pilot and feasibility trials. BMJ. 2016;355:i5239. 27777223 10.1136/bmj.i5239 PMC5076380

[27] Harris PA, Taylor R, Minor BL, Elliott V, Fernandez M, O’Neal L, et al. The REDCap consortium: building an international community of software platform partners. J Biomed Inform. 2019;95:103208. 31078660 10.1016/j.jbi.2019.103208 PMC7254481

[28] Harris PA, Taylor R, Thielke R, Payne J, Gonzalez N, Conde JG. Research electronic data capture (REDCap)—A metadata-driven methodology and workflow process for providing translational research informatics support. J Biomed Inform. 2009;42(2):377–81. 18929686 10.1016/j.jbi.2008.08.010 PMC2700030

[29] Walker BF, Hebert JJ, Stomski NJ, Clarke BR, Bowden RS, Losco B, et al. Outcomes of usual chiropractic. The OUCH randomized controlled trial of adverse events. LWW; 2013;1723-9. 10.1097/BRS.0b013e31829fefe4 23778372

[30] Viechtbauer W, Smits L, Kotz D, Budé L, Spigt M, Serroyen J, et al. A simple formula for the calculation of sample size in pilot studies. J Clin Epidemiol. 2015;68(11):1375–9. 26146089 10.1016/j.jclinepi.2015.04.014

[31] Gale NK, Heath G, Cameron E, Rashid S, Redwood S. Using the framework method for the analysis of qualitative data in multi-disciplinary health research. BMC Med Res Methodol. 2013;13(1):117. 24047204 10.1186/1471-2288-13-117 PMC3848812

[32] Abbott JH. The distinction between randomized clinical trials (RCTs) and preliminary feasibility and pilot studies: what they are and are not. J Orthop Sports Physical Ther. 2014;44:555–8. 10.2519/jospt.2014.0110 25082389

[33] Jenkins HJ, French SD, Young A, Moloney NA, Maher CG, Magnussen JS et al. Feasibility of testing the effectiveness of a theory-informed intervention to reduce imaging for low back pain: a pilot cluster randomised controlled trial. Pilot Feasibility Stud. 2022;8(1):249. 10.1186/s40814-022-01216-8 PMC9733261 36494716

[34] McKinn S, Bonner C, Jansen J, McCaffery K. Recruiting general practitioners as participants for qualitative and experimental primary care studies in Australia. Aust J Prim Health. 2015;21(3):354. 25074256 10.1071/PY14068

[35] Axén I, Björk Brämberg E, Galaasen Bakken A, Kwak L. Recruiting in intervention studies: challenges and solutions. BMJ Open. 2021;11(1):e044702. 33495262 10.1136/bmjopen-2020-044702 PMC7839899

[36] Axén I, Leboeuf-Yde C. Conducting practice-based projects among chiropractors: a manual. Chiropr Man Ther. 2013;21(1):8. 10.1186/2045-709X-21-8 PMC3577479 23369259

[37] Lalji R, Hofstetter L, Kongsted A, Von Wyl V, Braun J, Puhan MA, et al. Swiss chiropractic cohort (Swiss ChiCo) pilot study: feasibility for a musculoskeletal cohort study conducted within a nationwide practice-based research network. Eur Spine J. 2024;33(5):2068–78. 38480624 10.1007/s00586-024-08175-z

[38] Brown BT, Bonello R, Fernandez-Caamano R, Eaton S, Graham PL, Green H. Consumer characteristics and perceptions of chiropractic and chiropractic services in Australia: results from a cross-sectional survey. J Manip Physiol Ther. 2014;37(4):219–29. 10.1016/j.jmpt.2014.01.001 24679644

[39] Forsat ND, Palmowski A, Palmowski Y, Boers M, Buttgereit F. Recruitment and retention of older people in clinical research. Syst Lit Rev. 2020;68(12):2955–63. 10.1111/jgs.16875 33075140

[40] Kent P, Haines T, O’Sullivan P, Smith A, Campbell A, Schutze R, et al. Cognitive functional therapy with or without movement sensor biofeedback versus usual care for chronic, disabling low back pain (RESTORE): a randomised, controlled, three-arm, parallel group, phase 3, clinical trial. Lancet. 2023;401(10391):1866–77. 37146623 10.1016/S0140-6736(23)00441-5

[41] Pocovi NC, Lin C-WC, French SD, Graham PL, Van Dongen JM, Latimer J, et al. Effectiveness and cost-effectiveness of an individualised, progressive walking and education intervention for the prevention of low back pain recurrence in Australia (WalkBack): a randomised controlled trial. Lancet. 2024;404(10448):134–44. 38908392 10.1016/S0140-6736(24)00755-4

