# Supplementary material for: Does radiographic imaging for spinal pain in patients receiving chiropractic care change patient outcomes? A pilot randomised controlled trial
Source: Chiropr Man Therap. 2026 Jul 2;34:42. doi: 10.1186/s12998-026-00662-y (PMC13525698; doi:10.1186/s12998-026-00662-y)
Supplement: Supplementary file 1 [file 12998_2026_662_MOESM1_ESM.docx]

**Chiropractor post-study interview guide**

1. What was your experience of being involved in the study?
2. How did you find filling out the screening form?
   a) Did you complete it for all new patients? If not why/why not?
   b) Was the form something you could complete during the consult? Why/why not?
   c) Did you use a paper version or online version of the form? Would you continue to use it in that version if you were to enter a similar study?
3. Did you have any issues with identifying patients that were eligible to participate in the study? Why/why not?
4. What facilitated or challenged your ability to recruit participants for the study?
5. What was your experience in explaining the study to the patient and asking for their interest to participate?
6. Were you able to complete the patient follow-up surveys? Why/why not? Was there anything challenging about completing them? If so, what?
7. Were there any unanticipated advantages or disadvantages to being involved in the study?
8. Do you have any suggestions for improvement to the study design or recruitment process?
